# Real-world effectiveness and safety of nirmatrelvir-ritonavir (Paxlovid)-treated for COVID-19 patients with onset of more than 5 days: a retrospective cohort study

**DOI:** 10.3389/fphar.2024.1401658

**Published:** 2024-08-19

**Authors:** Ye Qiu, Hao Wen, Haoru Wang, Wenjun Sun, Guangchao Li, Shaoqiang Li, Yan Wang, Jingnan Zhai, Yangqing Zhan, Yutian Su, Zhiwei Long, Zhengtu Li, Feng Ye

**Affiliations:** ^1^ State Key Laboratory of Respiratory Disease, National Clinical Research Center for Respiratory Disease, Guangzhou Institute of Respiratory Health, The First Affiliated Hospital of Guangzhou Medical University, Guangzhou, China; ^2^ Gastroenterology and Respiratory Internal Medicine Department, Guangxi Medical University Cancer Hospital, Nanning, China; ^3^ First Clinical School, Guangzhou Medical University, Guangzhou, China

**Keywords:** COVID-19 patients, onset more than 5 days, Paxlovid, effectiveness and safety, viral load, virus elimination

## Abstract

**Background:**

Nirmatrelvir-ritonavir (Paxlovid) has received emergency use authorization from the US Food and Drug Administration owing to its effectiveness and safety. However, data on the effectiveness and safety of Paxlovid use in COVID-19 patients with onset of more than 5 days are lacking.

**Methods:**

A real-world retrospective study was performed during the outbreak involving the SARS-CoV-2 BA.5.2 subvariant. Hospitalized COVID-19 patients (including mild, moderate, severe and critical cases) were divided into three groups: Paxlovid treatment within (Group A) or more than (Group B) 5 days of COVID-19 onset and no Paxlovid treatment during more than 5 days of COVID-19 onset with only basic symptomatic treatment (Group C). Endpoints were all-cause 28-day mortality, improvement in clinical classification, and a composite endpoint of disease progression, viral load and virus elimination time. Safety was assessed by comparing adverse events reported during treatment in each group.

**Results:**

During the period, 248 hospitalized COVID-19 patients, including 55 in Group A, 170 in Group B, and 23 in Group C, were enrolled. There were no significant differences in the clinical classification improvement rate [80.0% (16/20) vs. 81.3% (52/64), *p* = 1.000; 60.0% (21/35) vs. 55.7% (59/106), *p* = 0.653, respectively] or all-cause 28-day mortality [0% (0/20) vs. 1.6% (1/64), *p* = 1.000; 11.4% (4/35) vs. 6.6% (7/106), *p* = 0.576, respectively] between Groups A and B for nonsevere and severe cases. However, the clinical classification improvement rate in Group B was markedly higher than that in Group C [81.3% (52/64) vs. 50.0% (6/12), *p* = 0.049] among nonsevere cases. Cycle threshold values of the N and ORF genes in Group B were significantly increased after Paxlovid treatment [31.14 (IQR 26.81–33.93) vs. 38.14 (IQR 36.92–40.00), *p* < 0.001; 31.33 (IQR 26.00–33.47) vs. 38.62 (IQR 35.62–40.00), *p* < 0.001, respectively]. No significant differences in reported adverse events of neurological disease (*p* = 0.571), liver injury (*p* = 0.960) or kidney injury (*p* = 0.193) between Group A and Group B were found.

**Conclusion:**

Paxlovid treatment within 10 days of onset can shorten the disease course of COVID-19 by reducing the viral load. Paxlovid is effective and safe in treating COVID-19 with onset of more than five or even 10 days when patients have a high viral load.

## 1 Introduction

Since 2019, the pandemic of coronavirus disease 2019 (COVID-19) has posed a serious threat to global health and placed an unprecedented strain on healthcare systems worldwide (Hammond et al., 2022; Najjar-Debbiny et al., 2023). Notably, COVID-19 continues to be a health concern. According to WHO data, there have been nearly 14 million newly confirmed cases worldwide from 27 March, 2023 to 20 December, 2023, including 74,281 deaths. Among these patients, 2,52,923 cases were confirmed in China (World Health Organization (WHO)). Thus, COVID-19 remains an important global infectious disease that deserves close attention.

Paxlovid contains nirmatrelvir [a severe acute respiratory syndrome coronavirus 2 (SARS-CoV-2) main protease inhibitor)] and ritonavir (a CYP3A4 inhibitor that reduces nirmatrelvir metabolism) and can effectively treat COVID-19 by inhibiting viral replication (Hammond et al., 2022; Dryden-Peterson et al., 2023; Najjar-Debbiny et al., 2023). Indeed, numerous studies have demonstrated that Paxlovid can significantly reduce the rate of hospitalization, intensive care unit admission, invasive mechanical ventilation usage, and mortality among patients with mild-to-moderate COVID-19 who are at risk of developing severe illness (Wong et al., 2022a; Wong et al., 2022b; Hammond et al., 2022; Dryden-Peterson et al., 2023; Lewnard et al., 2023; Najjar-Debbiny et al., 2023; Yang et al., 2023; Yip et al., 2023). Based on these studies, the protocol and guidelines for COVID-19 treatment recommend Paxlovid as being suitable for mild-moderate adult patients with a high risk of progression to severe disease within 5 days of COVID-19 onset and recommend only one course (5 days) (Hammond et al., 2022; Wong et al., 2022a; Wong et al., 2022b; Dryden-Peterson et al., 2023; Lewnard et al., 2023; Najjar-Debbiny et al., 2023; Yip et al., 2023; National Health Commission of the People’s Republic of China). However, we found that in the real world, the initial Paxlovid treatment time among a significant proportion of COVID-19 patients was more than 5 days after COVID-19 onset, yet data on the effectiveness and safety of Paxlovid use in COVID-19 patients with onset of more than 5 days are limited. Nevertheless, one small-sample study suggested that Paxlovid may reduce 28-day mortality rates in critical patients with invasive mechanical ventilation when the SARS-CoV-2 infection duration exceeds 5 days (Yang et al., 2023), and a matched observational cohort study showed that the effectiveness of Paxlovid treatment may decline for a treatment course dispensed ≥6 days after symptom onset or for patients who were not experiencing acute clinical symptoms (Lewnard et al., 2023). Thus, real-world evidence is urgently needed to ascertain the efficacy and safety of Paxlovid in treating patients with COVID-19 onset of more than 5 days.

In this real-world retrospective observational study, we enrolled hospitalized COVID-19 patients and explored the effectiveness and safety of Paxlovid use in those with onset of more than 5 days by comparing and analyzing clinical outcomes and virological indexes in those with Paxlovid treatment within or more than 5 days of COVID-19 onset and in those not treated with Paxlovid at more than 5 days after COVID-19 onset and only basic symptomatic treatment. In addition, we sought to comprehend the effectiveness and safety of multiple cycles of Paxlovid therapy. Finally, we aimed to provide evidence-based medicine for clinical management of patients with onset of COVID-19 of more than 5 days.

## 2 Methods

### 2.1 Diagnosis and clinical classification of COVID-19

The COVID-19 Diagnosis and Treatment Plan (trial version 10), as issued by the Ministry of Health in China (National Health Commission of the People’s Republic of China), was utilized for diagnosis and clinical classification of COVID-19 patients. COVID-19 was confirmed for patients with positive results by high-throughput metagenomic sequencing or real-time Reverse Transcription-Polymerase Chain Reaction (RT‒PCR) assays of nasal and pharyngeal swabs. For clinical classification, cases involving with only upper respiratory tract infections were deemed mild, while those involving persistent high fever (>3 days) and cough yet with a respiratory rate of less than 30 breaths/min and an oxygen saturation higher than 93% on room air at sea level were classified as moderate. Patients who had an oxygen saturation ≤93% on room air at sea level, a ratio of arterial partial pressure of oxygen to fraction of inspired oxygen ≤300 mmHg, or a respiratory rate ≥30 breaths/min were considered to have severe illness. Those who had respiratory failure requiring mechanical ventilation or who were in shock or had multiorgan failure requiring ICU care were considered to have critical disease (National Health Commission of the People’s Republic of China).

### 2.2 Study population and design

This real-world retrospective observational study enrolled patients with confirm1ed COVID-19 between 30 November, 2022 and 31 January, 2023, during the outbreak of the SARS-CoV-2 BA.5.2 subvariant in Guangzhou and hospitalized in the First Affiliated Hospital of Guangzhou Medical University. The earliest day that COVID-19 symptoms were experienced for patients was defined as the onset of COVID-19, and the hospitalized COVID-19 patients were divided into three groups: Paxlovid treatment within (Group A) or more than (Group B) 5 days of COVID-19 onset and those not treated with Paxlovid at more than 5 days after COVID-19 onset and only basic symptomatic treatment (Group C). The majority of patients in Group C declined Paxlovid treatment due to financial constraints and concerns about the high cost of the medication. The severity of their condition was not a factor in whether they received Paxlovid. Group B was further divided into Group B1 (6–10 days) and Group B2 (more than 10 days) based on a Paxlovid treatment time of within or more than 10 days of COVID-19 onset. The patients in all groups were classified into either a severe (severe or critical COVID-19) group or a nonsevere (mild or moderate COVID-19) group according to their initial clinical COVID-19 classification.

### 2.3 Enrollment and exclusion

Eligible hospitalized patients aged 18 years or older with a confirmed diagnosis of COVID-19 and complete information on physical indications, comorbidities, clinical symptoms, COVID-19 treatment information, and specific effectiveness were enrolled. Exclusion criteria included patients younger than 18 years, those with incomplete core clinical data or those who had used antivirals other than Paxlovid, such as ambavizumab/romisivir, lopinavir/ritonavir, remdesivir, molnupiravir and azvudine.

### 2.4 Data sources

We collected demographic characteristics, hospital admission and discharge data, registered death data, clinical symptoms, oxygen therapy data, inflammatory cytokine data, drug dispensing records, procedures, and laboratory tests from the hospital information system and entered the data into a predefined information collection sheet. When core information was missing, patients or their relatives were contacted by telephone for completion. The clinical data were collected, entered, and verified by two independent researchers for cross-checking to ensure data reliability.

### 2.5 Endpoints and safety

The primary endpoints were all-cause 28-day mortality and improvement of clinical classification. Improvement of clinical classification was defined as a demonstration of effectiveness when comparing the clinical classification after each treatment cycle with that before treatment. Clinical recovery in the nonsevere group and conversion to nonsevere COVID-19 or clinical recovery in the severe group were defined as clinical classification improvement. Maintenance of severe or critical COVID-19 in the severe group and conversion to severe or critical COVID-19 in the nonsevere group were defined as no improvement in clinical classification.

The secondary endpoints were a composite endpoint of disease progression (in-hospital mortality, intensive care unit admission, invasive mechanical ventilation use, oxygen therapy changes and length of hospitalization after administration of Paxlovid) (Hammond et al., 2022; Wong et al., 2022a; Dryden-Peterson et al., 2023), viral load and virus elimination time. During disease progression, changes in oxygen therapy to high levels included an increase in oxygen flow or high-flow nasal cannula oxygen therapy, and noninvasive positive pressure ventilation changed to invasive mechanical ventilation. Additionally, based on the COVID-19 Diagnosis and Treatment Plan (trial version 10) (National Health Commission of the People’s Republic of China), discharge was permitted if the patient’s condition had improved significantly, vital signs were stable, body temperature had been normalized for more than 24 h, and acute exudative lesions had improved significantly on lung imaging and if the patient could be switched to oral medication, with two consecutive negative nucleic acid results (two 24 h intervals) and no complications requiring further management. Safety was assessed by recording and comparing the adverse events reported during treatment in each group (Hammond et al., 2022).

### 2.6 Subgroup analysis

We divided the subgroup of patients with COVID-19 onset for more than 5 days (Group B) into Group B1 (treated at the time of COVID-19 onset for 6–10 days) and Group B2 (treated at the time of COVID-19 onset for more than 10 days). In the viral load subgroup, the Wilcoxon rank-sum test was used to compare the Cycle threshold (Ct) value measured by RT‒PCR from nasal and pharyngeal swabs before and after Paxlovid treatment to assess the effect of Paxlovid in lowering viral load. For virus elimination time, we counted the time from COVID-19 onset to two consecutive days of Ct values ≥35 for both the N and ORF genes by RT‒PCR at least 24 h apart (Lu et al., 2022; Wang et al., 2023) and compared them by the Wilcoxon rank-sum test.

### 2.7 Statistical analysis

Categorical variables are presented as the number and percentage of cases; continuous variables are displayed as the mean (SD) and median (25th and 75th quartile). Independent samples t tests, Wilcoxon rank-sum tests, Pearson chi-square tests, and Fisher’s exact tests were used to calculate differences in continuous and categorical variables between groups to ensure that the groups were balanced at baseline. All statistical analyses were performed with IBM SPSS Statistic (version 26.0). All significance tests were two-tailed, and a *p* value less than 0.05 was considered to indicate statistical significance.

## 3 Results

### 3.1 Baseline demographics and clinical characteristics

**FIGURE 1:**
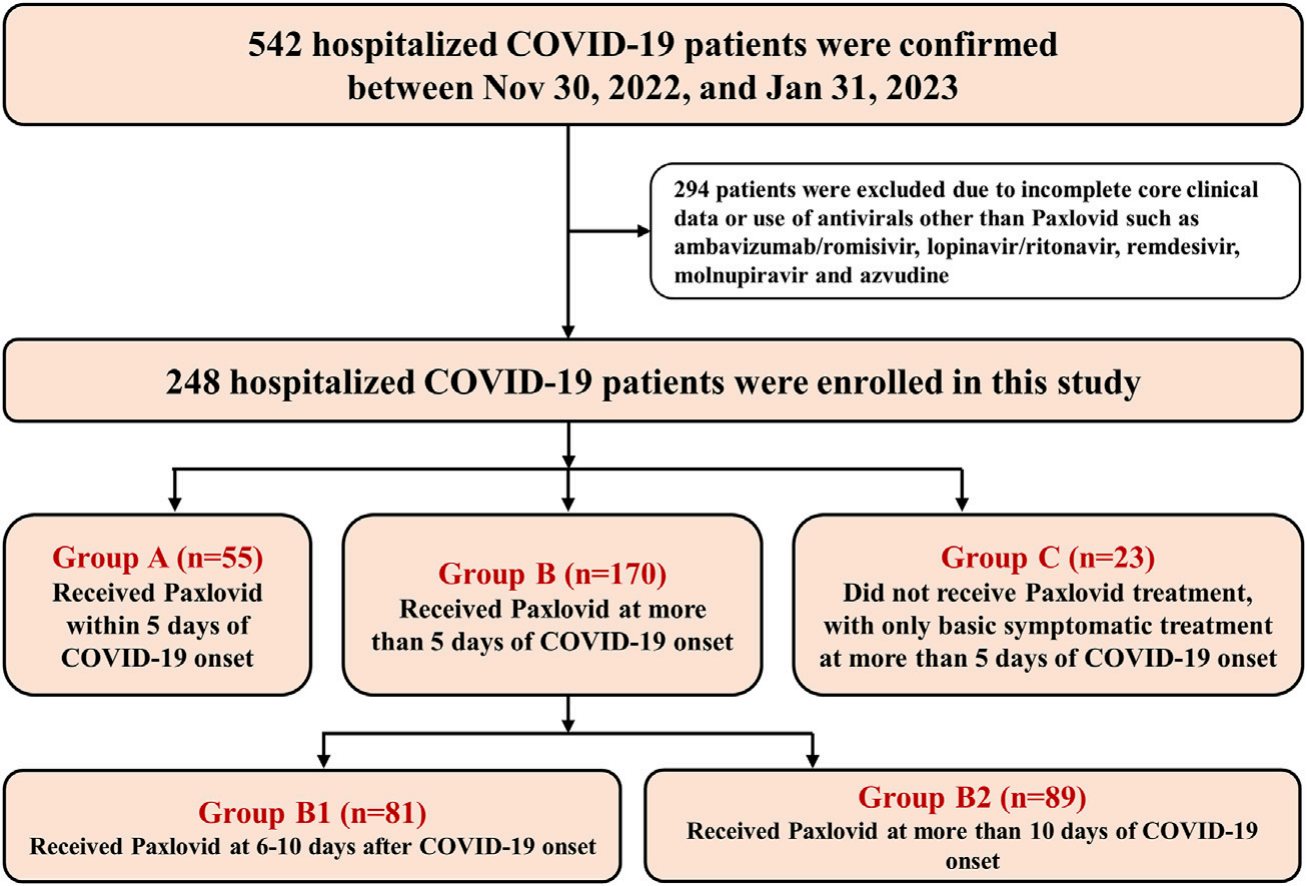
Flowchart of patient selection.

From 30 November, 2022 to 31 January, 2023, a total of 542 patients were confirmed to have COVID-19 and hospitalized in our hospital. After excluding 294 patients for incomplete core clinical information and/or those who had used antivirals other than Paxlovid, a total of 248 patients were enrolled and analyzed in this study, including 55 patients in Group A, 170 patients in Group B and 23 patients in Group C (Figure 1). The median times for receiving Paxlovid treatment in Groups A and B were 3 days (IQR 2–4 days) and 11 days (IQR 8–16 days), respectively.

In this retrospective observational study, for all 248 eligible patients, the mean age was 71.1 ± 13.6 years, and the body mass index (BMI) was 23.1 ± 4.2 kg/square meter. A total of 71.8% (178/248) were males. The most common risk factor for progression to severe COVID-19 in this study was age of 66 years or older (70.2%, 174/248), followed by chronic lung disease (46.0%, 114/248), cardiovascular disease (29.8%, 74/248), diabetes mellitus (27.8%, 69/248) and severe smoking (23.4%, 58/248). There were more patients in the severe (severe or critical COVID-19) group (61.3%, 152/248) than in the nonsevere (mild or moderate COVID-19) group (38.7%, 96/248). Among Groups A, B and C, the demographic characteristics, inflammatory factors and initial clinical classification of COVID-19 were well balanced. More information on the demographics and clinical characteristics of the cohort is provided in Table 1.

**TABLE 1:** Baseline characteristics of the full-analysis population.

	Group A (N = 55)	Group B (N = 170)	Group C (N = 23)	*p* value	
				A vs. B	B vs. C
Age, years					
Median (IQR)	69 (60–78)	72 (64–82)	72 (68–79)	0.177	0.807
By category				0.224	0.099
18–65	21 (38.2%)	50 (29.4%)	3 (13.0%)		
≥66	34 (61.8%)	120 (70.6%)	20 (87.0%)		
Sex				0.182	0.333
Male	36 (65.5%)	127 (74.7%)	15 (65.2%)		
Female	19 (34.5%)	43 (25.3%)	8 (34.8%)		
Body mass index					
Mean ± SD	23.9 ± 3.6	22.7 ± 4.5	23.2 ± 1.4	0.131	0.817
COVID-19 clinical classification			0.915	0.915	0.516
Mild	2 (3.6%)	10 (5.9%)	1 (4.3%)		
Moderate	18 (32.7%)	54 (31.8%)	11 (47.8%)		
Severe	27 (49.1%)	84 (49.4%)	9 (39.1%)		
Critical	8 (14.5%)	22 (12.9%)	2 (8.7%)		
Risk factors for severe illness from COVID-19					
Diabetes mellitus	17 (30.9%)	46 (27.1%)	6 (26.1%)	0.580	0.921
Cardiovascular disease	21 (38.2%)	43 (25.3%)	10 (43.5%)	0.066	0.067
Chronic lung disease	30 (54.5%)	73 (42.9%)	11 (47.8%)	0.133	0.657
Chronic liver disease	5 (9.1%)	21 (12.4%)	2 (8.7%)	0.511	0.869
Chronic kidney disease	5 (9.1%)	29 (17.1%)	4 (17.4%)	0.152	1.000
Immunodeficiency disease/immunosuppressive	7 (12.7%)	17 (10.0%)	1 (4.3%)	0.569	0.622
Severe smoking	12 (21.8%)	42 (24.7%)	2 (8.7%)	0.663	0.086
Inflammatory factor, median (IQR)					
Procalcitonin, (ng/mL)	0.07 (0.00–0.20)	0.05 (0.00–0.18)	0.06 (0.00–0.20)	0.510	0.624
D-Dimer, (ng/mL)	1,039 (567–2,234)	1,052 (630–2,353)	1,415 (637–3,262)	0.702	0.680
Interleukin- 6, (pg/mL)	14.1 (5.7–41.0)	9.9 (4.4–38.8)	7.9 (3.1–37.1)	0.682	0.505
C-reaction protein, (mg/dL)	2.87 (1.81–6.14)	3.15 (1.25–9.37)	1.13 (0.25–7.75)	0.914	0.172
Erythrocyte sedimentation rate, (mm/h)	55 (18–97)	47 (27–78)	51 (33–82)	0.594	0.573

Data are N (%), unless otherwise indicated. IQR, inter-quartile range; SD, standard deviation.

### 3.2 Effectiveness and safety

#### 3.2.1 Comparison among those receiving paxlovid within or more than 5 days of COVID-19 onset

Regarding the primary endpoints, there were no significant differences in the clinical classification improvement rate [80.0% (16/20) vs. 81.3% (52/64), *p* = 1.000; 60.0% (21/35) vs. 55.7% (59/106), *p* = 0.653, respectively] and all-cause 28-day mortality [0% (0/20) vs. 1.6% (1/64), *p* = 1.000; 11.4% (4/35) vs. 6.6% (7/106), *p* = 0.576, respectively] between Groups A and B for nonsevere and severe cases. For the secondary endpoints, there were no significant differences in the median length of hospitalization [9 (IQR: 6–16) vs. 8 (IQR: 6–13) days, *p* = 0.532], in-hospital mortality [7.3% (4/55) vs. 10.0% (17/170), *p* = 0.546], intensive care unit admission rate [3.6% (2/55) vs. 3.5% (6/170), *p* = 1.000] or invasive mechanical ventilation rate [5.5% (3/55) vs. 5.9% (10/170), *p* = 1.000] of the patients in Groups A and B. There was also no significant difference in oxygen therapy changes [14.5% (8/55) vs. 14.1% (24/170), *p* = 0.937] in the patients in Groups A and B or in the incidence of complications (acute respiratory distress syndrome, embolism and myocarditis) occurring during Paxlovid treatment in Groups A and B (*p* = 0.936, *p* = 0.830, *p* = 0.554) (Table 2).

**TABLE 2:** Comparison of the effectiveness and safety of receiving Paxlovid before and after 5 days of COVID-19 onset.

	Group A (N = 55)	Group B (N = 170)	*p* value
Effectiveness			
Primary endpoints			
Nonsevere group, N	20	64	
All-cause 28-day mortality	0 (0)	1 (1.6%)	1.000
Clinical classification improvement	16 (80.0%)	52 (81.3%)	1.000
Severe group, N	35	106	
All-cause 28-day mortality	4 (11.4%)	7 (6.6%)	0.576
Clinical classification improvement	21 (60.0%)	59 (55.7%)	0.653
Secondary endpoints			0.287
Length of hospitalization after the Paxlovid treatment, median (IQR)	9 (6–16)	8 (6–13)	0.532
In-hospital mortality	4 (7.3%)	17 (10.0%)	0.546
Intensive care unit admission	2 (3.6%)	6 (3.5%)	1.000
Invasive mechanical ventilation use	3 (5.5%)	10 (5.9%)	1.000
Oxygen therapy changes	8 (14.5%)	24 (14.1%)	0.937
Complications occurring during the Paxlovid treatment			0.603
Acute respiratory distress syndrome	4 (7.3%)	15 (8.8%)	0.936
Embolism	4 (7.3%)	9 (5.3%)	0.830
Myocarditis	2 (3.6%)	12 (7.1%)	0.554
Safety			
Poison and side effect			0.736
Neurological diseases	1 (1.8%)	2 (1.2%)	0.571
Liver injury	4 (7.3%)	10 (5.9%)	0.960
Kidney injury	5 (9.1%)	6 (3.5%)	0.193

Data are N (%), unless otherwise indicated. IQR, inter-quartile range.

Adverse events reported during treatment in Groups A and B were compared to evaluate the safety of Paxlovid in treating patients within or more than 5 days of COVID-19 onset, with no significant differences in neurological disease (*p* = 0.571), liver injury (*p* = 0.960) or kidney injury (*p* = 0.193) (Table 2).

#### 3.2.2 Comparison among patients with or without paxlovid treatment at more than 5 days of COVID-19 onset

The clinical effectiveness of Paxlovid treatment for patients with COVID-19 onset for more than 5 days was evaluated by comparing the difference in endpoints between Groups B and C. For the primary endpoints, there were no significant differences in all-cause 28-day mortality in nonsevere cases between Group B and Group C [1.6% (1/64) vs. 0% (0/12), *p* = 1.000]. However, a significant difference was observed in the rate of achieved clinical classification improvement between Group B and Group C among the nonsevere cases [81.3% (52/64) vs. 50.0% (6/12), *p* = 0.049]. In severe cases, there were no significant differences in the clinical classification improvement rate [55.7% (59/106) vs. 45.5% (5/12), *p* = 0.742] or all-cause 28-day mortality [6.6% (7/106) vs. 9.1% (1/12), *p* = 1.000] between Groups B and C.

For the secondary endpoints, in-hospital mortality, intensive care unit admission, invasive mechanical ventilation rate and oxygen therapy changes were 10.0% (17/170), 3.5% (6/170), 5.9% (10/170), 14.1% (24/170) vs. 8.7% (2/23), 8.7% (2/23), 13.0% (3/23), and 17.4% (4/23) in Groups B and C, respectively, none of which showed significant differences (*p* = 1.000, *p* = 0.542, *p* = 0.399, *p* = 0.918) (Table 3).

**TABLE 3:** Comparison of the effectiveness of or without Paxlovid treatment in patients at more than 5 days of COVID-19 onset.

	Group B (N = 170)	Group C (N = 23)	*p* value
Effectiveness			
Primary endpoints			
Nonsevere group, N	64	12	
All-cause 28-day mortality	1 (1.6%)	0 (0)	1.000
Clinical classification improvement	52 (81.3%)	6 (50.0%)	**0.049**
Severe group, N	106	11	
All-cause 28-day mortality	7 (6.6%)	1 (9.1%)	1.000
Clinical classification improvement	59 (55.7%)	5 (45.5%)	0.742
Secondary endpoints			0.829
In-hospital mortality	17 (10.0%)	2 (8.7%)	1.000
Intensive care unit admission	6 (3.5%)	2 (8.7%)	0.542
Invasive mechanical ventilation use	10 (5.9%)	3 (13.0%)	0.399
Oxygen therapy changes	24 (14.1%)	4 (17.4%)	0.918

Data are N (%), unless otherwise indicated. Bold values indicate data which have statistically different (*p* < 0.05).

To further explore and clarify the effective opportunity time of Paxlovid therapy in patients at more than 5 days of COVID-19 onset, we subdivided Group B into Group B1 and Group B2 based on the Paxlovid treatment time within and more than 10 days of COVID-19 onset. The median times for receiving Paxlovid treatment in Groups B1 and B2 were 8 days (IQR 7–9 days) and 16 days (IQR 13–22 days), respectively. The characteristics of the analysis population at baseline were balanced before comparing differences in endpoints among Groups B1, B2 and C.

For the primary endpoints, there were no significant differences in all-cause 28-day mortality between Groups B1 and B2, Groups B2 and C, or Groups B1 and C (*p* > 0.05) among nonsevere and severe cases. Similarly, there were no significant differences in the rate of achieved clinical classification improvement between Groups B1 and B2 and Groups B1 and C (*p* > 0.05) among nonsevere cases and between Groups B2 and C and Groups B1 and C (*p* > 0.05) among severe cases. However, in nonsevere cases, the rate of achieved clinical classification improvement in Group B2 was higher than that in Group C [87.9% (29/33) vs. 50.0% (6/12), p = 0.022]. And in severe cases, the rate of achieved clinical classification improvement in Group B1 was higher than that in Group B2 [66.0% (33/50) vs. 46.4% (26/56), p = 0.043] (Table 4).

**TABLE 4:** Subgroup of patients with COVID-19 onset of more than 5 days according to the time of onset to treatment.

	Group B (N = 170)		Group C (N = 23)	*p* value		
	Group B1 (6–10 days) (N = 81)	Group B2 (≥11 days) (N = 89)				
Age, years						
Median (IQR)	71 (63–81)	73 (65–83)	72 (68–79)	0.849		
By category				0.141		
18–65	27 (33.3%)	23 (25.8%)	3 (13.0%)			
≥66	54 (66.7%)	66 (74.2%)	20 (87.0%)			
Sex				0.430		
Male	63 (77.8%)	64 (71.9%)	15 (65.2%)			
Female	18 (22.2%)	25 (28.1%)	8 (34.8%)			
Body mass index						
Mean ± SD	22.8 ± 4.6	22.6 ± 4.4	23.2 ± 1.4	0.758		
COVID-19 clinical classification				0.276		
Mild	6 (7.4%)	4 (4.5%)	1 (4.3%)			
Moderate	25 (30.9%)	29 (32.6%)	11 (47.8%)			
Severe	44 (54.3%)	40 (44.9%)	9 (39.1%)			
Critical	6 (7.4%)	16 (18.0%)	2 (8.7%)			
Risk factors for severe illness from COVID-19						
Diabetes mellitus	23 (28.4%)	23 (25.8%)	6 (26.1%)	0.928		
Cardiovascular disease	18 (22.2%)	25 (28.1%)	10 (43.5%)	0.129		
Chronic lung disease	37 (45.7%)	36 (40.4%)	11 (47.8%)	0.716		
Chronic liver disease	11 (13.6%)	10 (11.2%)	2 (8.7%)	0.781		
Chronic kidney disease	14 (17.3%)	15 (16.9%)	4 (17.4%)	0.996		
Immunosuppressive disease or immunosuppressive	5 (6.2%)	12 (13.5%)	1 (4.3%)	0.173		
Severe smoking	19 (23.5%)	23 (25.8%)	2 (8.7%)	0.214		
Effectiveness						
Primary endpoints						
Nonsevere group, N	31	33	12			
All-cause 28-day mortality	0 (0)	1 (3.0%)	0 (0)	1.000*	1.000^†^	1.000^‡^
Clinical classification improvement	23 (74.2%)	29 (87.9%)	6 (50.0%)	0.161*	**0.022** ^†^	0.248^‡^
Severe group, N	50	56	11			
All-cause 28-day mortality	5 (10.0%)	2 (3.6%)	1 (9.1%)	0.251*	0.421^†^	1.000^‡^
Clinical classification improvement	33 (66.0%)	26 (46.4%)	5 (45.5%)	0.043*	0.953^†^	0.353^‡^
Secondary endpoints						
In-hospital mortality	6 (7.4%)	11 (12.4%)	2 (8.7%)	0.282*	0.901^†^	1.000^‡^
Intensive care unit admission	2 (2.5%)	4 (4.5%)	2 (8.7%)	0.684*	0.601^†^	0.211^‡^
Invasive mechanical ventilation use	3 (3.7%)	7 (7.9%)	3 (13.0%)	0.409*	0.714^†^	0.120^‡^
Oxygen therapy changes	13 (16.0%)	11 (12.4%)	4 (17.4%)	0.490*	0.773^†^	1.000^‡^

Data are N (%), unless otherwise indicated. IQR, inter-quartile range; SD, standard deviation. *p values were calculated between groups B1 and B2, ^†^ *p* values were calculated between groups B2 and C, ^‡^ *p* values were calculated between groups B1 and C. Bold values indicate data which have statistically different (*p* < 0.05).

#### 3.2.3 Analysis of the viral load of patients treated with paxlovid

The viral load subgroup was established after excluding 128 patients in Group B missing viral load information who were admitted on the basis of a positive rapid antigen test and high viral load was defined as Ct values < 35 (National Health Commission of the People’s Republic of China). Finally, 42 patients were included in the subgroup, including 17 patients in Group B1 and 25 in Group B2. The viral load of nucleocapsid protein gene (N gene) and open reading frame gene (ORF gene) in these 42 patients within 4 days after Paxlovid treatment showed a significant reduction [median Ct values: 38.62 (IQR 35.88–40.00) vs. 31.01 (IQR 27.31–33.53), *p* < 0.001; 39.19 (IQR 35.46–40.00) vs. 31.41 (IQR 26.25–33.35), *p* < 0.001, respectively].

Seventeen COVID-19 patients treated with Paxlovid 6–10 days (in Group B1) after COVID-19 onset showed a significant reduction in the viral load of the N and ORF genes [median Ct values: 40.00 (34.11–40.00) vs. 30.61 (29.72–31.99), *p* = 0.006; 40.00 (33.72–40.00) vs. 31.67 (30.22–32.48), *p* = 0.040, respectively].

**FIGURE 2:**
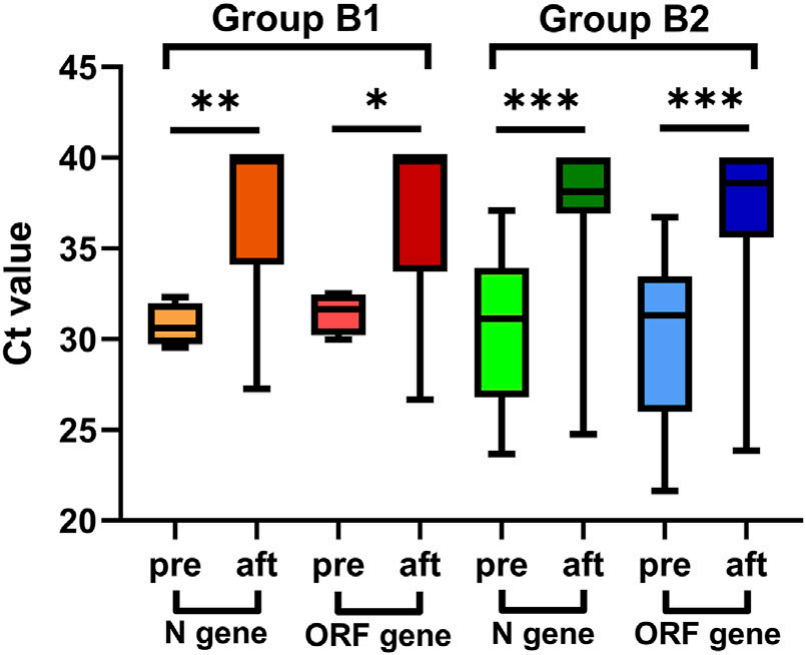
Viral load of patients treated with Paxlovid at more than five days after COVID-19 onset, pre: before Paxlovid treatment, aft: after Paxlovid treatment.

Twenty-five COVID-19 patients treated with Paxlovid ≥10 days (in Group B2) after COVID-19 onset showed a significant reduction in the viral load of the N and ORF genes [median Ct values: 38.14 (36.92–40.00) vs. 31.14 (26.81–33.93), *p* < 0.001; 38.62 (35.62–40.00) vs. 31.33 (IQR 26.00–33.47), *p* < 0.001, respectively] (Figure 2).

Additionally, for the N gene, the median Ct values were 8.34 (IQR 3.96–13.08) and 6.29 (IQR 2.40–10.34) for patients in Group B1 and Group B2, respectively, with no significance (*p* = 0.454). For the ORF gene, the medians were 7.55 (IQR 3.67–8.71) and 6.31 (IQR 2.16–9.43) for patients in Group B1 and Group B2, respectively (*p* = 0.539) (Table 5).

**TABLE 5:** Subgroup of patients treated with Paxlovid at more than 5 days after COVID-19 onset regarding the cycle threshold value.

	Cycle threshold value before treatment, median (IQR)	Cycle threshold value after treatment, median (IQR)	Increment of Ct values, median (IQR)	*p* value	
All patients treated with Paxlovid after 5 days of COVID-19 onset (Group B)					
Nucleocapsid protein-encoding gene	31.01 (27.31–33.53)	38.62 (35.88–40.00)		<0.001*	
Open reading frame gene	31.41 (26.25–33.35)	39.19 (35.46–40.00)		<0.001*	
Patients treated 6–10 days after COVID-19 onset (Group B1)					
Nucleocapsid protein-encoding gene	30.61 (29.72–31.99)	40.00 (34.11–40.00)	8.34 (3.96–10.08)	0.006*	0.635^†^
Open reading frame gene	31.67 (30.22–32.48)	40.00 (33.72–40.00)	7.55 (3.67–8.71)	0.040*	0.539^†^
Patients treated more than 10 days after COVID-19 onset (Group B2)					
Nucleocapsid protein-encoding gene	31.14 (26.81–33.93)	38.14 (36.92–40.00)	6.29 (2.40–10.34)	<0.001*	
Open reading frame gene	31.33 (26.00–33.47)	38.62 (35.62–40.00)	6.31 (2.16–9.43)	<0.001*	

IQR, inter-quartile range. *p values were calculated of Ct values before and after Paxlovid treatment, ^†^ *p* values represented the probability that there is no difference in the increment of Ct values between patients in groups B1 and B2. Bold values indicate data which have statistically different (*p* < 0.05).

#### 3.2.4 Analysis of the virus elimination time of patients treated with paxlovid

**FIGURE 3:**
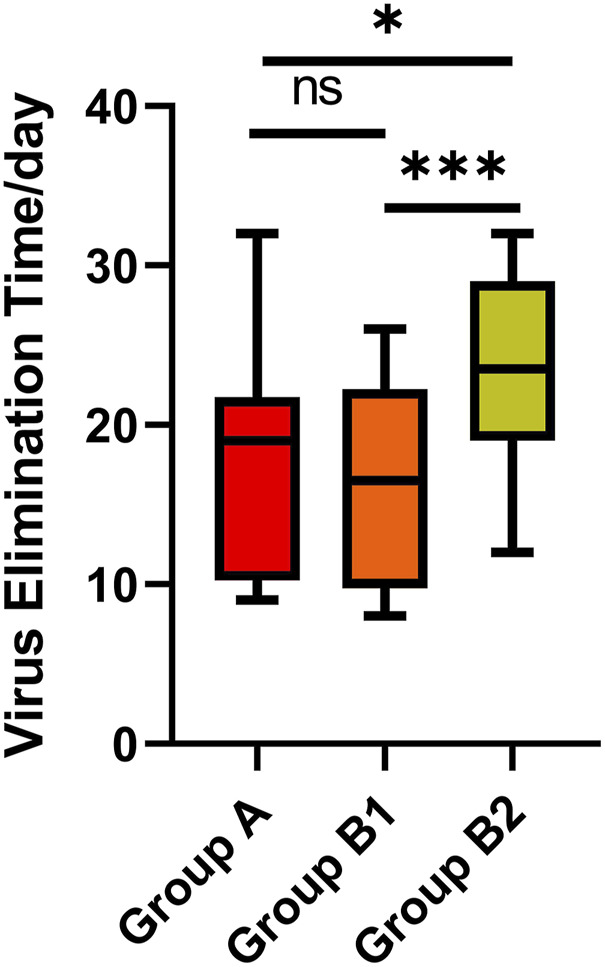
The virus elimination time of patients treated with Paxlovid.

Virus elimination was defined as two consecutive negative results (Ct values ≥ 35 for both the N and ORF genes by RT‒PCR) at least 24 h apart (Lu et al., 2022; Wang et al., 2023). Viral elimination time was defined as from COVID-19 onset to the date of the first negative test of consecutive negative results. We included eight patients in Group A, 18 patients in Group B1 and 30 patients in Group B2 who had an exact virus elimination time of less than 35 days, reducing the impact of extreme values. There was no significant difference in virus elimination time between Groups A and B1, and the medians were 19.0 (IQR 10.3–21.8) and 16.5 (IQR 9.8–22.3), respectively (*p* = 0.724). However, the time in Group B2 (median 23.5, IQR 19.0–29.0) was longer than that in Groups A and B1 (*p* = 0.045, *p* = 0.001, respectively), which indicates that receiving Paxlovid treatment within 10 days of COVID-19 onset had a better effect of shortening the virus elimination time than receiving it at more than 10 days after onset (Table 6) (Figure 3).

**TABLE 6:** Subgroup of patients treated with Paxlovid regarding virus elimination time.

	Group A	Group B		*p* value		
	(N = 8)	Group B1 (6–10 days) (N = 18)	Group B2 (≥11 days) (N = 30)			
Virus elimination time, median (IQR)	19.0 (10.3–21.8)	16.5 (9.8–22.3)	23.5 (19.0–29.0)	0.724*	**0.001** ^†^	**0.045** ^‡^

IQR, inter-quartile range. *p value was calculated between groups A and B1, ^†^ *p* value was calculated between groups B1 and B2, ^‡^ *p* value was calculated between groups A and B2. Bold values indicate data which have statistically different (*p* < 0.05).

#### 3.2.5 Patients with multiple cycles of paxlovid treatment

This subgroup was established based on the number of cycles of Paxlovid treatment that patients received. We describe the clinical classification rate of patients after receiving different cycles of treatment. The rate of achieved clinical classification improvement was 65.8% (148 of 225) in patients treated with one cycle of Paxlovid treatment. For patients whose viral load remained high after a single cycle of treatment and without significant toxicity or side effects, after obtaining consent, we administered two or more cycles of Paxlovid. In 75% (12 of 16), clinical classification improved after two cycles of Paxlovid treatment, and 25% (1 of 4) of patients improved after three or more cycles (Table 7).

**TABLE 7:** Subgroup of patients with multiple cycles of Paxlovid treatment.

	Paxlovid single cycle treatment (N = 225)	Two cycles of Paxlovid treatment (N = 16)	Three and more cycles of Paxlovid treatment (N = 4)
Patients with improvement in clinical classification	148 (65.8%)	12 (75.0%)	1 (25.0%)
Patients with no improvement in clinical classification	77 (34.2%)	4 (25.0%)	3 (75.0%)

Data are N (%), unless otherwise indicated.

## 4 Discussion

In this retrospective observational study, we evaluated the effectiveness and safety of Paxlovid in treating patients with COVID-19 onset of more than 5 days. Our study demonstrates that Paxlovid treatment is effective and safe for COVID-19 patients with onset of more than 5 days, especially for those with nonsevere cases or within 10 days of onset. Paxlovid reduced the risk of conversion from mild and moderate illness to severe and critical illness and also helped severe and critical cases convert to nonsevere cases. In addition, Paxlovid can significantly reduce viral load in patients with COVID-19 and shorten the duration of illness by shortening the virus elimination time. Paxlovid is not as effective in treating patients with severe disease who have been ill for more than 10 days. Therefore, we recommend that Paxlovid treatment should be received as soon as possible after the onset of COVID-19 and that it is appropriate to be received within 10 days of onset. In addition, our small-sample study suggests that patients who remained clinically symptomatic after a single cycle of treatment, with have a high viral load and able to tolerate Paxlovid might benefit from multiple cycles of Paxlovid treatment, but the exact effectiveness and safety need to be further determined in larger-sample studies. Our study provides a valuable rationale and experience for clinical Paxlovid treatment in patients with COVID-19 onset of more than 5 days.

The role of Paxlovid is to prevent the virus from replicating and reproducing at an early stage, reducing the incidence of severe and critical illness (Hammond et al., 2022; Najjar-Debbiny et al., 2023). However, each patient’s immune function is different, the ability to clear virus-infected cells differs, and viruses entering cells are cleared at different times, resulting in different times of viral replication and spread (Primorac et al., 2022). After the same time from the onset of the disease, different patients will have different viral loads. Previous studies have shown that viral RNA in throat swabs from rhesus macaques was still detectable above the lower limit of detection at 9 days after virus infection, suggesting that SARS-CoV-2 replicates for a long time *in vivo* (Salguero et al., 2021). In our study, we also found that the virus continued to replicate for up to 36 days after the onset of disease in patients, which provides a rationale for use of Paxlovid to stop viral replication after COVID-19 onset of more than 5 days.

Current studies on Paxlovid are basically related to treatment within 5 days of onset and a single cycle of treatment, whereas studies and data on the effectiveness and safety of Paxlovid use in COVID-19 patients with onset of more than 5 days and multiple cycles of treatment are lacking. However, in real-world treatment, it has been observed that a large proportion of patients are unable to receive Paxlovid treatment within 5 days of COVID-19 onset due to cascading referrals or other factors; hence, our study has important clinical implications. In our retrospective observational study, Paxlovid was proven to reduce viral load in patients with onset of COVID-19 of more than 5 days, which was a contributing factor for the effectiveness of Paxlovid in treating SARS-CoV-2-infected patients at onset time of over 5 days. For this reason, it is more scientific to analyze viral load (measured Ct value) to guide use of Paxlovid than to classify patients based on the number of days since the onset of the disease alone. Furthermore, our study shows that Paxlovid treatment can be used not only for treatment of severe cases 5 days after COVID-19 onset but also for nonsevere cases at 5 days after onset to reduce the risk of severe disease.

Additionally, we found that the ability of Paxlovid treatment to improve clinical classification and shorten the virus elimination time in severe and critical cases with COVID-19 onset of more than 10 days was poorer than that achieved within 10 days, which may be explained by the life cycle of SARS-CoV-2. Previous studies have demonstrated an increasing and then decreasing viral load trend of SARS-CoV-2 after invasion *in vivo*, with peak levels early in infection (Salguero et al., 2021), which may result in a limited effect of Paxlovid in reducing viral load in patients with COVID-19 onset of more than 10 days.

This study also has some limitations. First, this was a single-center study, the patients included were mainly concentrated in the southern region of China, and the time span was small. As the variant strain was predominantly Omicron BA5.2 (Chinese Center for Disease Control and Prevention), the effect of Paxlovid on other strains was not assessed. Second, the study included only hospitalized patients, which is not representative of the demographic characteristics of all patients with COVID-19. Finally, the small number of patients in our study who did not use antiviral drugs made it difficult for some of the chi-square tests to show significant results, and thus, further refinement is needed in future studies.

## 5 Conclusion

The effectiveness and safety of Paxlovid in treating patients at more than 5 days and within 10 days of COVID-19 onset is the same as treating patients within 5 days of onset. Paxlovid may be recommended for treatment even with COVID-19 onset more than 5 days if the viral load is high, especially in patients with onset of less than 10 days. In addition, multiple cycles of Paxlovid treatment can have some degree of clinical effectiveness in patients who remain clinically symptomatic after a single cycle of treatment, still have a high viral load and tolerate Paxlovid.

## Data Availability

The original contributions presented in the study are included in the article/supplementary material, further inquiries can be directed to the corresponding authors.

## Abbreviations

COVID-19, coronavirus disease 2019; Ct, cycle threshold; N gene, nucleocapsid protein gene; ORF gene, open reading frame gene; RT‒PCR, reverse transcription-polymerase chain reaction; SARS-CoV-2, severe acute respiratory syndrome coronavirus 2.
